# The mitochondrial genome of the grape powdery mildew pathogen *Erysiphe necator* is intron rich and exhibits a distinct gene organization

**DOI:** 10.1038/s41598-021-93481-5

**Published:** 2021-07-06

**Authors:** Alex Z. Zaccaron, Jorge T. De Souza, Ioannis Stergiopoulos

**Affiliations:** 1 Department of Plant Pathology, University of California Davis, One Shields Avenue, Davis, CA 95616-8751 USA; 2 Present Address: Department of Plant Pathology, Federal University of Lavras (UFLA), Lavras, MG 37200-000 Brazil

**Keywords:** Fungi, Pathogens

## Abstract

Powdery mildews are notorious fungal plant pathogens but only limited information exists on their genomes. Here we present the mitochondrial genome of the grape powdery mildew fungus *Erysiphe necator* and a high-quality mitochondrial gene annotation generated through cloning and Sanger sequencing of full-length cDNA clones. The *E. necator* mitochondrial genome consists of a circular DNA sequence of 188,577 bp that harbors a core set of 14 protein-coding genes that are typically present in fungal mitochondrial genomes, along with genes encoding the small and large ribosomal subunits, a ribosomal protein S3, and 25 mitochondrial-encoded transfer RNAs (mt-tRNAs). Interestingly, it also exhibits a distinct gene organization with atypical bicistronic-like expression of the *nad4L*/*nad5* and *atp6*/*nad3* gene pairs, and contains a large number of 70 introns, making it one of the richest in introns mitochondrial genomes among fungi. Sixty-four intronic ORFs were also found, most of which encoded homing endonucleases of the LAGLIDADG or GIY-YIG families. Further comparative analysis of five *E. necator* isolates revealed 203 polymorphic sites, but only five were located within exons of the core mitochondrial genes. These results provide insights into the organization of mitochondrial genomes of powdery mildews and represent valuable resources for population genetic and evolutionary studies.

## Introduction

*Erysiphe necator* (syn. *Uncinula necator*) is an obligate biotrophic ascomycete fungus that belongs to the Erysiphaceae family (Leotiomycetes; Erysiphales) and causes grape powdery mildew, one of the most widespread and destructive fungal diseases in vineyards across the world^1^. The predicted 126 ± 18 Mb nuclear genome of *E. necator* was sequenced before from five isolates of the fungus that originated from organic vineyards (i.e., isolates Branching and e1 − 101) or fields that received regular fungicide applications for control of the pathogen (i.e., isolates C-strain, Lodi, and Ranch9)^2^. The analysis revealed a highly repetitive genome with frequent structural variations among the isolates that likely play a role in the adaptive responses of the fungus to fungicide stress. However, apart from this study, genomic resources for *E. necator* are to this date relatively scarce and there is no public reference mitochondrial (mt) genome available for this pathogen.

Mitochondria are double-membrane bound organelles commonly recognized as the power factories of eukaryotic cells, due to their ability to produce energy through oxidative phosphorylation^3,4^. They carry their own genomes that are contained within single circular chromosomes. In mammals, mt genomes are approximately 16.6 kb in length and contain genes that typically lack introns^5,6^. In contrast, fungal mt genomes vary remarkably in size, ranging from 12 kb in *Rozella allomycis*^7^ to 272 kb in *Morchella importuna*^8^, and harbor genes that may too show extensive variation in intron content.

In fungi, mt genomes contain a standard set of 14 core genes (i.e., *atp6*, *atp8, atp9*, *nad1*, *nad2*, *nad3*, *nad4*, *nad4L*, *nad5, nad6, cob, cox1*, *cox2,* and *cox3*) that encode proteins involved in the electron transport chain (ETC) and oxidative phosphorylation^9^. They also harbor two genes encoding the small and large ribosomal subunits (*rns* and *rnl*, respectively) and a set of mt-encoded transfer RNAs (mt-tRNAs). Two additional genes, *rps3* and *rnpB*, that code for the 40S ribosomal protein S3 and the RNA subunit of the mitochondrial RNase P, respectively, are also sporadically found in fungal mt genomes^9^. Although most fungi exhibit a relatively similar repertoire of mt genes, in contrast the order of these genes is usually not well conserved, even among species of the same genus^10^. Nonetheless, some commonalities in gene arrangements exist as well, as for example is the case for the gene pairs *nad4L*/*nad5* and *nad2*/*nad3*, which appear next to each other in the mt genomes of most fungal species^11^.

Fungal mt genes also exhibit large variation in their intron numbers, which in some species may be completely absent, as for example in the wheat pathogen *Zymoseptoria tritici*^12^, while in others there might be as many as 80 introns, as for example in the ‘blue-stain’ fungus of conifers *Endoconidiophora resinifera*^13^. In general, fungal mt introns are typically classified into group I and group II^14^, with group I introns further being classified into seven subgroups, i.e., IA, IA3, IB, IC1, IC2, ID, and I derived (I*)^15^. In contrast to spliceosomal introns, group I and group II introns resemble mobile genetic elements and often harbor open reading frames (ORFs) encoding catalytic enzymes that enable intron self-splicing and transposition to an intronless cognate allele. In particular, group I mt introns typically contain ORFs that encode homing endonucleases (HEs) of the LAGLIDADG or GIY-YIG families, whereas group II introns typically encode reverse transcriptases (RTs). Although both group I and group II introns can be found in fungi, the majority of fungal mt introns are of group I and have been shown to exhibit extensive presence/absence variation, owing to their mobility and horizontal mode of transmission^15^.

In this study, we present a comprehensive assembly and annotation of the mt genome of *E. necator* isolate C-strain. All core protein-coding genes had their annotation manually curated by cDNA cloning and sequencing, which rectified spurious mitochondrial gene annotations that were not resolved by RNA-seq data alone. The results herein provide further insights into mt genome organization within Erysiphales and constitute valuable genomic resources for powdery mildew pathogens.

## Results

### Assembly and general features of the *E. necator* mt genome

BLASTn searches with the mt genome of *Sclerotinia borealis* (NC_025200.1) against the nuclear genome assembly of *E. necator* C-strain returned an 188,576 bp long scaffold (JNVN01000008.1) that represented the mt genome of *E. necator.* The scaffold contained homologs of all core mt genes, whereas the first and last 56 bp overlapped 100%, suggesting circularity. One 153 bp gap was present at 282 bp from one of the ends of the scaffold, but it was patched with whole-genome sequencing reads of *E. necator* C-strain (SRR1448449), thus generating a gapless mt genome assembly.

**Figure 1:**
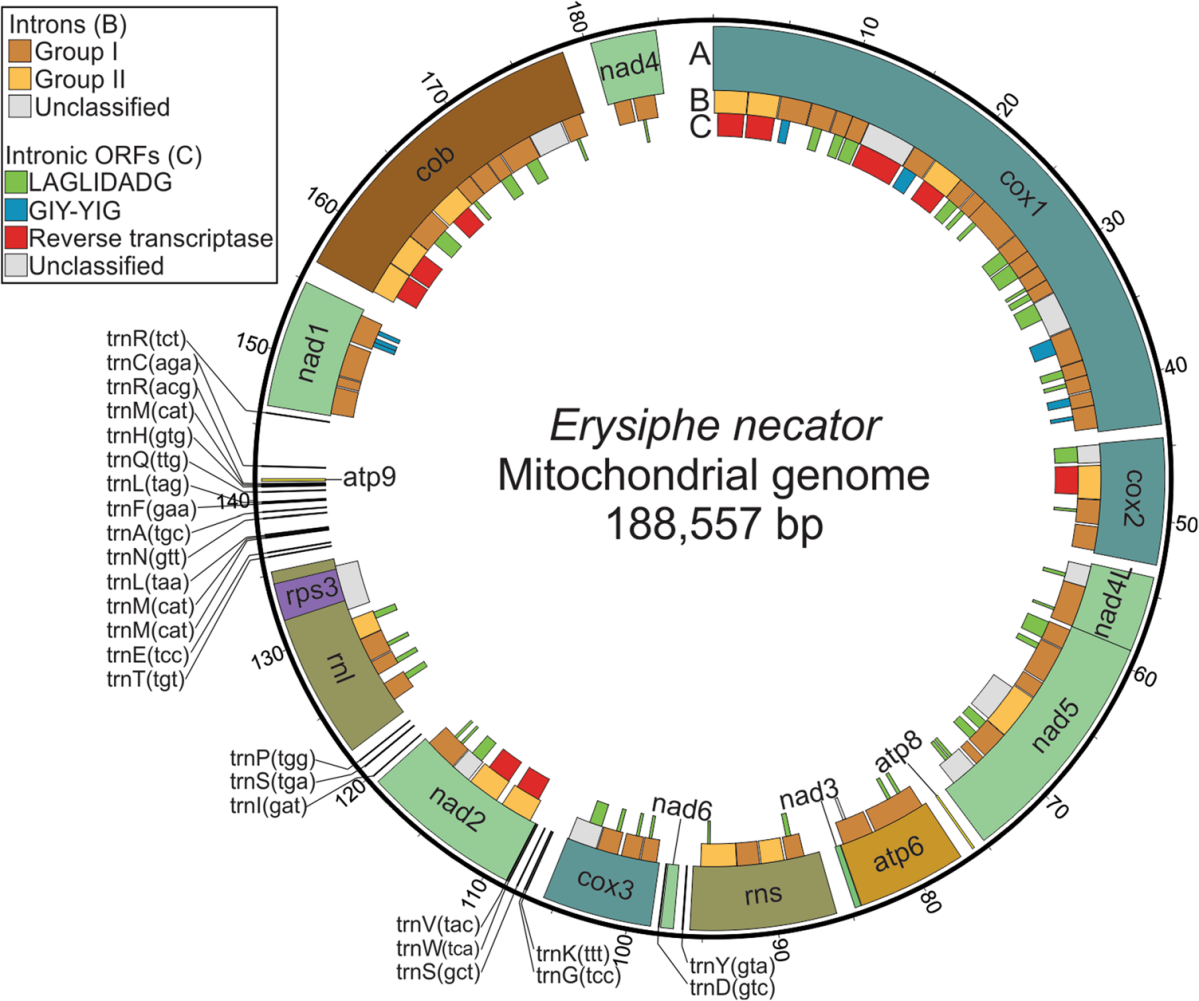
Organization of the mitochondrial (mt) genome of the grape powdery mildew fungus *Erysiphe necator*. The *E. necator* mt genome is a long and circular DNA molecule of 188,577 bp in size. Tracks: (**A**) Core protein-coding and other conserved genes present in the mt genome of *E. necator*. These include genes encoding the subunits of complex I (*nad1*, *nad2*, *nad3*, *nad4*, *nad4L*, *nad5* and *nad6*), complex III (*cob*), complex IV (*cox1*, *cox2* and *cox3*), the ATP-synthase complex (*atp6*, *atp8* and *atp9*), the small and large ribosomal subunits (*rns* and *rnl*), the ribosomal protein S3 (*rps3*), and a set of mt-tRNAs. (**B**) Introns present in the mt genes of *E. necator*. The introns are classified as group I and group II, or as unclassified. (**C**) Open reading frames (ORFs) present within introns, encoding homing endonucleases of the LAGLIDADG or GIY-YIG families, or reverse transcriptase. The figure was created with Circos v0.69-8^58^ (http://www.circos.ca) and further edited with Inkscape v1.0.2 (https://inkscape.org).

The resulting mt genome of *E. necator* C-strain corresponded to a long, circular, gapless DNA sequence containing 188,577 bp (Fig. 1, Table 1). The overall GC content was 33.8%, which is on the high-end for a fungal mt genome (Supplementary Fig. S1, Supplementary Table S1). The GC content of the protein-coding mt genes was 29.4%, reflecting preference for AT-rich codons (Supplementary Table S2), whereas the GC content of intergenic regions and introns was 38.9% and 33.1%, respectively, indicating that they largely contribute to the overall high GC content of the *E. necator* mt genome. GC-skew [(G − C)/(G + C)] and AT-skew [(A − T)/(A + T)] values were both positive (0.101 and 0.031, respectively), which is highly unusual as a positive AT-skew is rather rare in fungal mt genomes and thus far has been reported only in *Scytalidium auriculariicola* among 16 members of the Leotiomycetes^16^.

**Table 1:** Assembly and gene annotation statistics of the mitochondrial genome of *Erysiphe necator*.

Feature	Value
Total size (bp)	188,577
Intergenic regions size (bp)	28,343
Intronic regions size (bp)	139,477
Overall GC (%)	33.8
Core coding sequences GC (%)	29.4
Intergenic regions GC (%)	38.9
Intronic regions GC (%)	33.1
GC-skew (G − C)/(G + C)	0.101
AT-skew (A − T)/(A + T)	0.031
Repetitive DNA (%)	8.0
Genes	106
Introns	70
Intronic ORFs	64
LAGLIDADG ORFs	44
GIY-YIG ORFS	9
Reverse transcriptase ORFs	10

A total of 106 genes and other ORFs were predicted in the mt genome of *E. necator*, all of which are transcribed from the sense strand. Coding-sequences of the mt core genes accounted for 8.3% (15.8 kb) of the genome, whereas intergenic regions and introns covered 15.0% (28.3 kb) and 73.9% (139.5 kb) of the genome, respectively, thus contributing to its enlargement. A self-blast search further revealed a considerable amount of repetitive DNA, which accounted for 8.0% of the mt genome (Table 1). In total, 104 forward, 23 palindromic, and 11 reverse short exact repeats were identified (Supplementary Table S3). Forty-five short tandem repeats were also identified, most of which were concentrated within intergenic or intronic regions (Supplementary Table S4). A notable exception was the tandem repeat ATCCGTAGG, which encoded for Ser-Val-Gly (SVG) and was inserted seven consecutive times in-frame with the last exon of *nad2*. This indicates that next to their potential role in genome rearrangements^17–20^, tandem repeats in the mt genome of *E. necator* actively contribute to the modification of protein sequences.

### Gene content and organization of the *E. necator* mt genome

**Figure 2:**
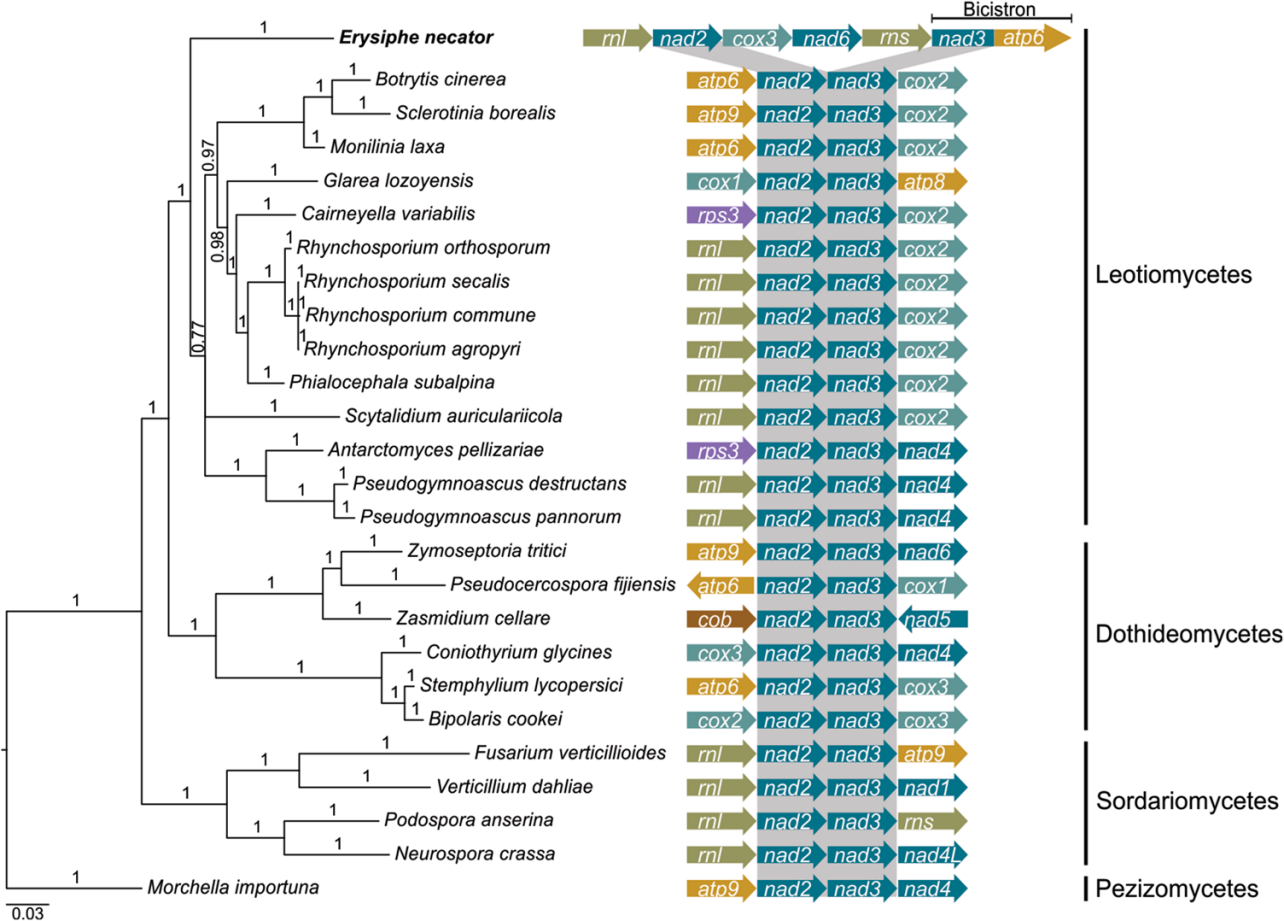
The mitochondrial (mt) genome of *Erysiphe necator* has an atypical organization of the *nad2* and *nad3* genes. A Bayesian phylogenetic tree of the mt genomes of *E. necator* and 25 other Ascomycetes is shown on the left-hand side of the image. The phylogenetic tree was inferred with MrBayes based on the concatenated alignment of the protein sequences of 12 mt genes (*atp6*, *nad1*-*6*, *nad4L*, *cox1*-*3,* and *cob*). For comparison, a phylogenetic tree based on the nuclear genomes is shown in Supplementary Fig. S8. Supporting values of branches are indicated as Bayesian posterior probabilities. *Morchella importuna* was used as outgroup. On the right-hand side of the image, the gene organization on each side of the *nad2* and *nad3* genes is indicted for the species shown in the tree. Genes are represented as arrows and are shown in the same order as they appear in the mt genomes. The *nad2* and *nad3* genes are typically next to each other in fungal mt genomes but not in the mt genome of *E. necator,* in which *nad3* and *atp6* are instead expressed bicistronically in the same RNA transcript. Accession numbers of the sequences utilized to construct the tree are available in Supplementary Table S12. The phylogenetic tree was edited with FigTree v1.4.2^66^ (http://tree.bio.ed.ac.uk/software/figtree/) and the figure was created with Inkscape v1.0.2 (https://inkscape.org).

The ab initio gene predictions performed with MFannot revealed that all the 14 core mt protein-coding genes are single-copy. The rRNA genes, *rns* and *rnl,* were also predicted within the mt genome, whereas *rps3*, which codes for the ribosomal protein S3, was detected within the fifth intron of *rnl* (*rnl*-i5). However, *rnpB*, which encodes the RNA subunit of the mt RNase P, was absent (Fig. 1, Table 2). As frequently observed in fungal mt genomes, *nad5* and *nad4L* were located next to each other in the mt genome of *E. necator*. In contrast, *nad2* and *nad3*, which are commonly arranged side-by-side (Fig. 2), were 29.9 kb apart and separated by the presence of three genes between them, namely *rns*, *nad6,* and *cox3* (Fig. 1). Moreover, instead of clustering with *nad2*, *nad3* clustered with *atp6*, from which it was separated by a short intergenic region of 44 bp. Collectively, these observations indicate that *E. necator* has a unique arrangement of mt genes compared to non-powdery mildew fungal species.

**Table 2:** Overall statistics of the core mitochondrial genes of *Erysiphe necator*.

Gene	Length (bp)	No. of introns	Exonic region (bp)	Intronic region (bp)	Intronic region (%)	Intron density	Start codon	Stop codon
*atp6*	7339	2	756	6583	89.7	2.6	ATG	TAA
*atp8*	147	0	147	0	0	0	ATG	TAG
*atp9*	180	0	180	0	0	0	TTA	TAG
*nad1*	8585	4	1062	7523	87.6	3.8	ATG	TAA
*nad2*	10,819	4	1755	9064	83.8	2.3	ATG	TAG
*nad3*	417	0	417	0	0	0	ATG	TAG
*nad4*	4420	2	1443	2977	67.4	1.4	ATG	TAG
*nad4L*	5097	2	270	4827	94.7	7.4	ATG	TAA
*nad5*	16,147	7	1959	14,188	87.9	3.6	ATG	TAG
*nad6*	852	0	852	0	0	0	ATG	TAG
*cox1*	43,512	22	1608	41,904	96.3	13.7	ATG	TAG
*cox2*	8485	4	756	7729	91.1	5.3	ATG	TAA
*cox3*	7364	4	816	6548	88.9	4.9	ATG	TAA
*cob*	21,964	10	1170	20,794	94.7	8.5	ATG	TAG
*rps3*	2526	0	2526	0	0	0	ATG	TAA
*rnl*	13,111	5	3497	9614	73.3	1.4	–	–
*rns*	9865	4	2139	7726	78.3	1.9	–	–

Intron density represents the number of introns per kb of exonic sequence.

Next to the core set of 14 protein-coding genes, 25 mt-tRNA genes, whose products are able to recognize the standard set of 20 amino acids required for the synthesis of the mt-encoded proteins, were also predicted within the *E. necator* mitogenome (Fig. 1). All predicted mt-tRNAs also fold into common cloverleaf-like secondary structures (Supplementary Fig. S2). Most mt-tRNA genes were single-copy, except for those encoding mt-tRNAs that decode arginine [*trnR(tct)* and *trnR(acg*)], leucine [*trnL(tag)* and *trnL(taa)*], and serine [*trnS(tga)* and *trnS(gct)*] that each had two copies, and the mt-tRNA gene for methionine [*trnM(cat)*] that was present in three copies. Almost all the mt-tRNA genes of *E. necator* were clustered in the vicinity of the *rnl* gene, a pattern that has been previously observed in fungal mt genomes and further positively correlates to the conservation of gene order^11,13,16,21^. Specifically, of the 25 mt-tRNA genes, 15 were located within a 9.6 kb mt-tRNA-rich region between *rnl* and *nad1,* three were present between *rnl* and *nad2,* whereas the remaining seven were located upstream of *rnl,* between *nad2* and *cox3* (*n* = 5), *cox3* and *nad6* (*n* = 1), and *nad6* and *rns* (*n* = 1) (Fig. 1).

### Sanger sequencing of full-length cDNA clones and identification of bicistronic genes

To annotate the mt genes of *E. necator*, the publicly available RNA-seq data (SRR1502871 to SRR1502882) that were previously used to assist the gene annotation of the *E. necator* nuclear genome^2^, was mapped to its mt genome. However, of the 393.3 million reads processed, only 2619 reads (0.0007%) mapped to the mt genome, with the majority (1604; 61.2%) aligning to the large or small ribosomal subunits. The rest of the genes sustained variable coverage with *nad3*, *nad4L*, *nad6*, *atp6*, and *atp8* having five or less reads mapping to their exons, thus prohibiting their accurate annotation. Therefore, an alternative approach was followed in order to properly curate the automatically inferred mt gene structures. Specifically, all 14 core protein-coding genes and the two ribosomal subunits were PCR amplified from a cDNA template of isolate C-strain and Sanger sequenced (Supplementary Fig. S3, Supplementary Table S5). In this way, all mt genes of *E. necator* had their structures manually inspected and successfully verified.

These experiments showed that all 14 protein-coding genes, the two rRNA genes, and the *rps3* gene present in the mt genome of *E. necator* were expressed. Of the 17 mt genes, eight had their in silico annotation confirmed by Sanger sequencing of their corresponding cDNA clones. These included the genes that were in silico annotated as intronless (i.e., *nad3*, *nad6*, *atp8*, *atp9,* and *rps3*) as well as *nad1*, *nad2,* and *nad5*. Interestingly, among the intronless genes whose ORFs were verified was *atp9,* even though it was predicted to encode a 59 amino acid long protein instead of the 74 amino acid atp9 protein typically found in other fungal species (Supplementary Table S6). A multiple sequence alignment of fungal atp9 proteins showed that the *E. necator* atp9 was missing 11 amino acids at its N-terminus and a few other amino acids in relatively well-conserved regions, suggesting that it might not be functional (Supplementary Fig. S4). However, a BLAST search against the nuclear genome of *E. necator* using the mt *atp9* as query revealed the presence of a 530 bp nuclear counterpart (KHJ33827), which encodes a 156 amino acid protein with a 76 amino acid long N-terminal mt targeting sequence. Evidence of allotropic expression of the nuclear *atp9* was observed based on RNA-seq data (Supplementary Fig. S5), indicating that, as in other fungi^13,22^, the nuclear *atp9* of *E. necator* could be a functional substitute of its truncated mt one.

Although eight mt genes had their in silico annotation verified, the remaining (i.e., *cob*, *cox1*, *cox2*, *cox3*, *atp6*, *nad4*, *nad4L*, *rns,* and *rnl*) needed to have their predicted gene models manually adjusted. A total of seven exons were missed by the in silico annotations, including four in *cox1*, two in *nad4L,* and one in *atp6*. Also, three predicted exons in *cob* were absent in the sequenced cDNA of this gene. Curation of *nad4L* extended its coding sequence until it overlapped with *nad5* by one base pair, in that the last nucleotide of *nad4L* stop codon (TAA) was also the first nucleotide of *nad5* start codon (ATG). By using primers located at the start codon of *nad4L* and at the stop codon of *nad5* (Supplementary Fig. S3, Supplementary Table S5), an RT-PCR assay showed that the ORFs of these two genes were co-transcribed as a single RNA transcript (Supplementary Fig. S6). Similar to the *nad4L*/*nad5* bicistron, *atp6* and *nad3* were also physically close to each other, and were co-transcribed in the same RNA transcript (Supplementary Fig. S6). The only gene present between the gene pairs *nad4L*/*nad5* and *atp6*/*nad3* was *atp8*, and thus transcription of *atp8* as a polycistronic unit was investigated. However, there was no evidence suggestive of co-transcription of *nad4L*/*nad5/atp8* or *atp8/atp6/nad3.*

### The mt genes of *E. necator* hold a large repertoire of introns and intron-encoded ORFs

The automatic gene annotations and subsequent manual curations revealed an unusually large number of 70 introns within the core mt genes of *E. necator,* with lengths varying from 714 bp (*nad1-*i2) to 4142 bp (*atp6-*i1). Among the core protein-coding and rRNA mt genes, five genes (i.e., *nad6*, *nad3*, *atp8*, *atp9*, and *rps3*) and all the mt-tRNAs were intronless, whereas the rest harbored from as few as two introns in *atp6* to as many as 22 introns in *cox1* (Fig. 1, Table 2). The large number of introns present in *cox1*, which accounted for 96.3% of its sequence, expanded the size of this gene to 43.5 kb, which is comparable to the 47.5 kb long *cox1* from *Endoconidiophora rosinifera*, the longest *cox1* reported to date among members of the Ascomycetes^13^. Intron density (i.e., the number of introns per kb of coding sequence) was also highest for *cox1* (13.7), followed by *cob* (8.5), and *nad4L* (7.4) (Table 2). This is perhaps not surprising, as *cox1* and *cob* are known to possess large intron numbers as compared to other fungal mt genes, and to exhibit frequent intron gain-and-loss events^15,23,24^.

As for most fungi, the majority (*n* = 63) of mt introns in *E. necator* resembled self-splicing introns, which based on their putative secondary structure could be classified as group I (*n* = 48) and group II (*n* = 13) introns. Group I introns were further classified into subgroups IB (*n* = 27), IC2 (*n* = 9), ID (*n* = 6), I derived (*n* = 4), IC1 (*n* = 1), and IA (*n* = 1) (Fig. 1). Notably, a set of 64 ORFs were found residing within the group I and II introns of the *E. necator* mt genes, of which 52 encoded HEs of the LAGLIDADG (*n* = 44) and GIY-YIG (*n* = 8) families, and ten encoded proteins with a domain architecture composed of an RT and an intron maturase. As expected, predicted HEs of the LAGLIDADG and GIY-YIG families were usually contained within group I introns, whereas RT-encoding ORFs were associated with group II introns (Supplementary Table S7). Specifically, of the 52 ORFs encoding HEs, 43 were located within group I introns, and nine RT-encoding ORFs were located within group II introns. However, exceptions were identified in the two ribosomal genes as, for example, *rns*-i4 and *rnl-*i4 contained ORFs with the LAGLIDADG nuclease motif, although they were classified as group II introns. Finally, of the 44 LAGLIDADG and eight GIY-YIG family HEs, 25 and five, respectively, appeared to have truncated domains, thus corresponding to likely degenerated HEs (Supplementary Table S8). The remaining two ORFs residing within the group I and II introns of the *E. necator* mt genes encoded a hybrid GIY-YIG/RT protein and a nuclease-associated modular DNA-binding domain 1 (NUMOD1), and were present within *nad5*-i4 and *atp6*-i2, respectively.

A notable feature of intronic HEs is that they can be inserted in-frame and thus translated as a fusion protein with their upstream exon. This, consequently, enhances their expression and their chances of fixation within a population^25^. In *E. necator*, a total of 58 ORFs encoding HEs or RTs were located within introns of protein-coding genes, of which 41 were in-frame with the upstream exon (Supplementary Table S8) and 25 further lacked stop codons in the region between the upstream exon and the predicted ORF start. Of these 25 ORFs, 14 encoded HEs of the LAGLIDADG family and four of the GIY-YIG family, whereas the remaining seven encoded RTs. Moreover, the 25 ORFs were overall closer to their upstream in-frame exons (average of 188 bp) compared to all the 64 intronic ORFs (average of 526 bp) found within the mt genes of *E. necator*. Collectively, these findings suggest that these 25 ORFs are likely capable of fusing with their in-frame upstream exons as a means of promoting their expression and fixation in the mt genome.

### Mt genomes are highly conserved among *E. necator* isolates

**Figure 3:**
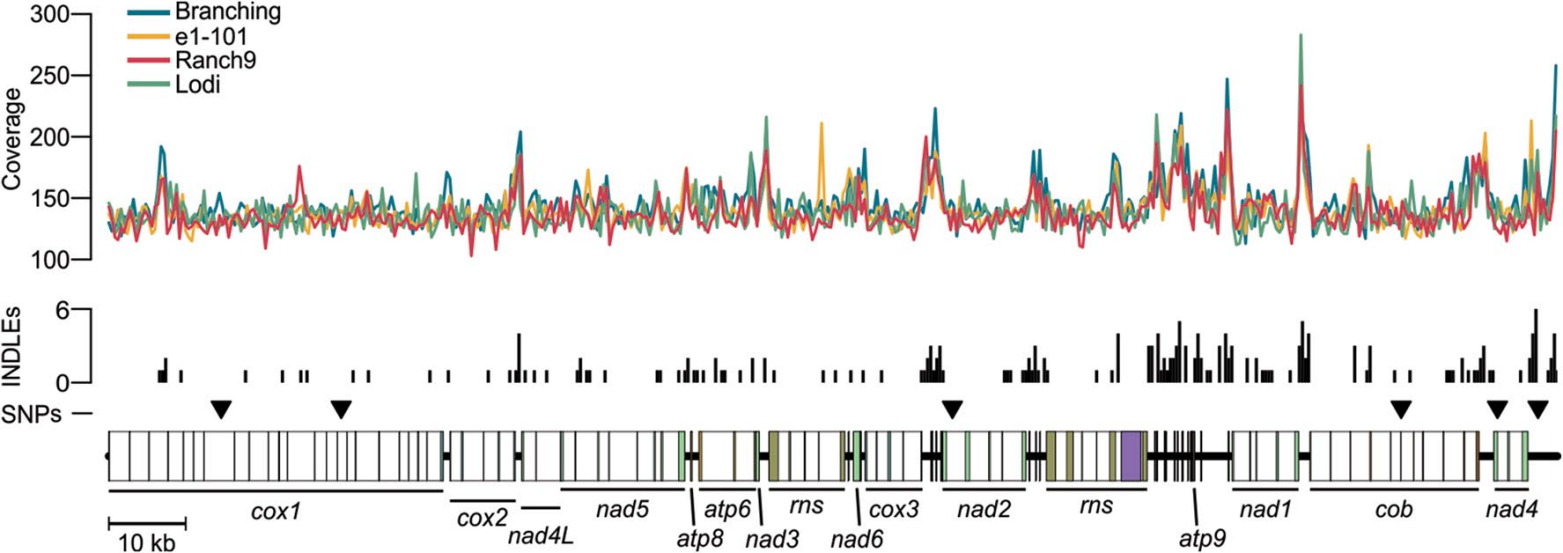
Comparative analysis of the mitochondrial (mt) genomes of five isolates of *E. necator* shows no presence/absence of introns and low genetic variability within functional regions. Whole-genome sequencing (WGS) coverage of four *E. necator* isolates across the reference mt genome of isolate C-strain is shown at the top. The histogram shows the number of insertions or deletions (INDELs) identified in different regions of the mt genome. Location of identified single nucleotide polymorphisms (SNPs) are indicated with triangles (total of six). The WGS coverage suggests that all regions of the reference mt genome are conserved in the other four isolates analyzed. Low number of SNPs and presence of almost all INDELs within intergenic or intronic regions indicate low genetic variability within functional regions of the mt genome of *E. necator*. Coverage of WGS reads and the INDEL histogram were generated with a non-overlapping sliding window of 400 bp. Coverage of WGS reads was normalized to 100 × prior alignment. The figure was generated with R v4.0.3 (https://www.r-project.org) and further edited with Inkscape v1.0.2 (https://inkscape.org).

By querying the mt genome of *E. necator* C-strain with BLASTn against the NCBI genomes database, scaffolds were identified that contained the mt genomes of isolates Branching (JNUS01000009.1), Ranch9 (JNUT01000020.1), and e1 − 101 (JOKO01000016.1). The mt genome of isolate Lodi was also identified but it parted into two scaffolds (JNUU01000038.1 and JNUU01000071.1). The size of the scaffolds containing the mt genome of the four isolates was comparable to that of isolate C-strain (188,577 bp), and ranged from 185,650 bp in Lodi, to 188,575 bp in Branching, 188,647 bp in e1 − 101, and 188,770 bp in Ranch9. Alignments with the mt genome of C-strain revealed a high level of conservation among the mt genomes of the five isolates. Specifically, all of the 106 genes and ORFs identified in the mt genome of C-strain were present in the mt genomes of the other four *E. necator* isolates. Also conserved was the order and orientation of these elements in the genome as well as the size and positions of intergenic regions and introns. The only exception appeared to be intron *nad5*-i4, which seemed absent in isolate Lodi (Supplementary Fig. S7). However, this intron was located at the breakpoint between the two scaffolds that contained the mt genome of this isolate, whereas by mapping the whole genome sequencing (WGS) reads from isolate Lodi to the mt genome of C-strain, *nad5*-i4 had normal coverage (Fig. 3), suggesting that it is also conserved in Lodi.

Although mt gene content and genome organization were fully conserved among the five isolates of *E. necator*, 203 polymorphic sites were identified (Supplementary Table S9). Of the 203 polymorphic sites, 197 were short insertions or deletions (INDELs) and only six were single-nucleotide polymorphisms (SNPs). INDELs were abundant within intergenic regions and introns, particularly in the mt-tRNA-rich region between *rns* and *nad1* (Fig. 3). From the 203 polymorphic sites, 126 were located within intergenic regions, 65 were present within introns, while 12 were located within exons of mt genes or intronic ORFs (Table 3, Supplementary Table S9). Among these 12 polymorphic sites, eight were short INDELs corresponding to microsatellite-like homopolymeric regions of eight or more consecutive nucleotides. Five of the INDELs caused frameshifts of LAGLIDADG-encoding ORFs within *cox1*-i11 [(C)_9–11_], *nad5*-i2 [(T)_10–11_ and (G)_8–11_], *cox3*-i2 [(G)_12–14_] and *cob*-i5 [(G)_8–12_], and two were located within *rns* [(G)_11–12_] and *rnl* [(G)_9–11_]. The remaining INDEL was identified within the last exon of *nad2* and corresponded to the tandem repeat ATCCGTAGG, which encoded for Ser-Val-Gly. This repeat was present seven times in isolates C-strain, Ranch9 and Lodi, and six times in isolates e1 − 101 and Branching (Table 3). Finally, of the remaining four polymorphic sites present within functional regions, two were located within intronic ORFs and two within coding sequences of conserved mt genes. Of the later ones, one induced a synonymous change at codon 151 of *nad4* (c.453C>A; p.V151V) and was found only in isolate Branching. However, the other one triggered a missense mutation at codon 143 (c.428G>C) of *cob*, which produces the notorious p.G143A amino acid substitution in cytochrome b that confers high levels of resistance to QoI fungicides^26^. This mutation was absent in C-strain, e1 − 101, and Branching but was present in isolates Ranch9 and Lodi (Table 3).

**Table 3:** Polymorphic sites within functional regions of the mitochondrial genome of *Erysiphe necator.*

Position (bp)	Gene/ORF	Variant (DNA)	Variant (protein)	C-strain	e1 − 101	Branching	Ranch9	Lodi
14,629	cox1-i7-RT	c.2094A>C	p.L698F	A	C	C	C	C
22,554	cox1-i11-LD	c.134C[11]; [10]; [9]	p.P48Lfs*10;p.P49Lfs*18	C[11]	C[9]	C[10]	C[10]	C[10]
61,221	nad5-i2-LD	c.34T[11];[10]	p.L15Yfs*5	T[11]	T[11]	T[11]	T[10]	T[10]
61,250	nad5-i2-LD	c.63G[10];[11];[8]	p.T25Dfs*16; p.G24Dfs*16	G[10]	G[8]	G[11]	G[10]	G[10]
86,443	*rns*	n.470G[11];[12]	–	G[11]	G[11]	G[11]	G[12]	G[12]
100,647	cox3-i2-LD	c.217G[14];[12]	p.G77*fs	G[14]	G[12]	G[14]	G[12]	G[12]
109,826	nad2-i1-RT	c.555C>T	p.L185L	C	C	C	T	T
119,171	*nad2*	c.1534TCCGTAGGA[7];[6]	p.SVG512[7];[6]	ATCCGTAGG[7]	ATCCGTAGG[6]	ATCCGTAGG[6]	ATCCGTAGG[7]	ATCCGTAGG[7]
130,655	*rnl*	n.2481G[9];[10];[11]	–	G[11]	G[10]	G[11]	G[9]	G[9]
167,386	cob-i5-LD	c.321G[12];[10];[11];[8]	p.G111Rfs*7;p.G111Afs*70;p.G110Afs*70	G[12]	G[11]	G[8]	G[10]	G[10]
168,253	*cob*	c.428G>C	p.G143A	G	G	G	C	C
180,789	*nad4*	c.453A>C	p.V151V	A	A	C	A	A

For each polymorphic site, its position in the genome is shown followed by the gene or intronic ORF affected by the polymorphism. The alleles for the respective isolates are shown. Variants at the DNA and protein levels are described according to the Human Genome Variation Society (HGVS) recommendations. Intronic ORFs encoding LAGLIDADG or reverse transcriptase domains are indicated with LD and RT, respectively.

**Figure 4:**
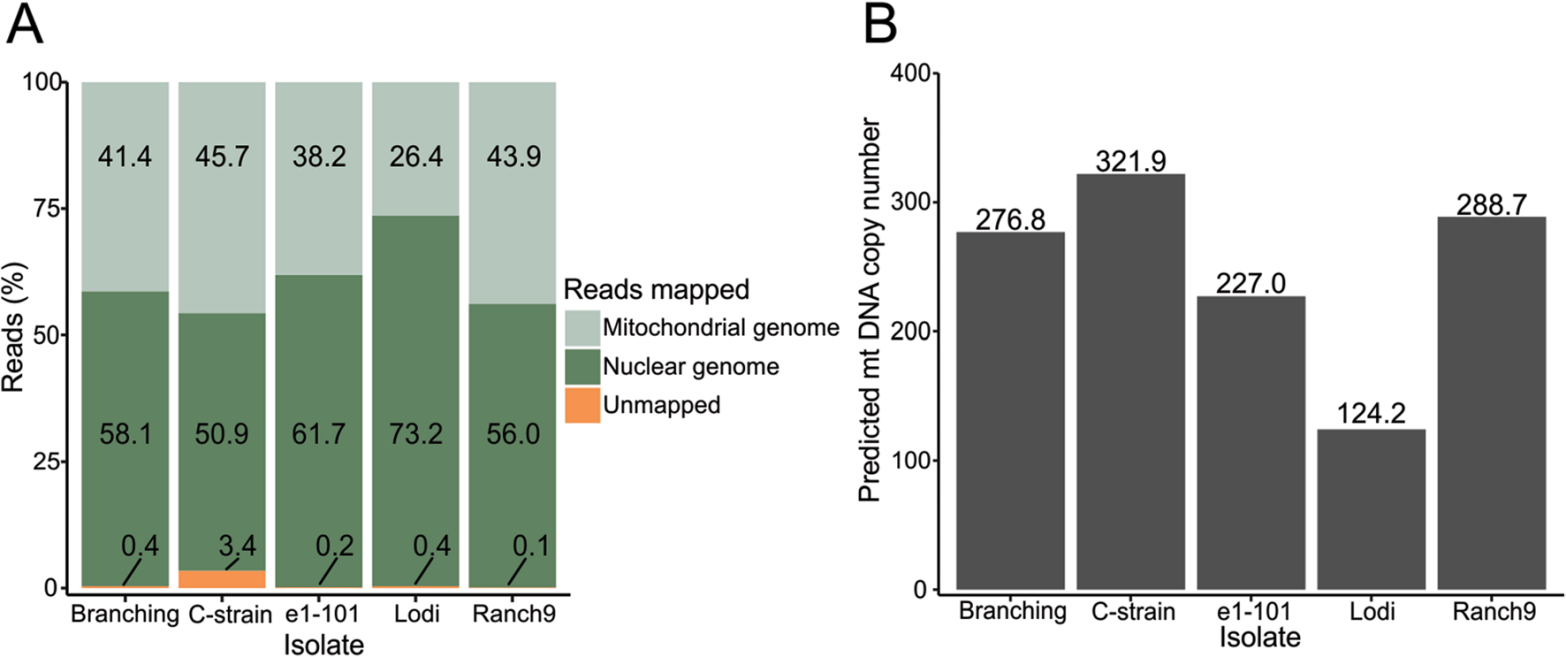
Estimated mitochondrial (mt) DNA copy numbers among isolates of *E. necator*. (**A**) Bar-plot showing the percentage of whole-genome sequencing (WGS) reads from five *E. necator* isolates mapped to the nuclear and mt genomes of *E. necator* C-strain. The large percentage of WGS reads mapped to the mt genome indicates high abundance of mt DNA compared to nuclear DNA. (**B**) Box-plot showing the estimated mt genome copy number per cell for the analyzed isolates. Mt genome copy number was calculated based on the ratio between the mt genome coverage and the nuclear genome coverage.

Interestingly, among the five *E. necator* isolates, 26% to 44% of the WGS reads mapped to the mt genome instead of the nuclear genome (Fig. 4A, Supplementary Table S10). This indicates that the mt genome is overrepresented in the sequenced reads, most likely as a result of the multi-copy nature of mitochondria in cells. Based on the mt genome coverage to nuclear genome coverage ratio, the estimated mitochondria copy number per cell varied from 124 to 322 (Fig. 4B, Supplementary Table S11). Finally, by dividing the sequenced base pairs from reads that did not map to the mt genome by the calculated coverage of the nuclear genome, then the estimated size of the nuclear genome of *E. necator* is between 78 and 95 Mbp (Supplementary Table S11). This estimate is considerably lower than the 126 ± 18 Mb genome size reckoned before using *k*-mer analysis^2^, indicating that the later approach might have overestimated the genome size of *E. necator*.

## Discussion

In this study, we present a high-quality mt genome for *E. necator*, an economically important powdery mildew pathogen, and thus provide further insights into the mt genome organization of members of the Erysiphales. Our analysis showed that the mt genome of *E. necator* is large but compact with dozens of group I introns encoding mostly HEs from the LAGLIDADG and GIY-YIG families. Moreover, the gene pairs *nad4L*/*nad5* and *atp6*/*nad3* exhibited bicistronic expression, which is exceptional among fungi. Further analysis of the mt genomes of five *E. necator* isolates revealed a high level of conservation of gene content and order but large variations in predicted mt DNA copy-numbers per isolate. Overall, the genomic resources presented herein will be of great value for future studies of population and evolutionary genomics of powdery mildews.

Identification of short exons is challenging as they can be easily mis-predicted by ab initio predictors. In this study, we followed a systematic approach to assemble the mt genome of *E. necator*, which consisted of validating the ab initio predictions of the mt genes by mapping of RNA-seq reads to the assembly and subsequent Sanger sequencing of full-length cDNA clones. This approach allowed us to correct several erroneous predictions that were not resolved by RNA-seq alone, and thus accurately adjust the gene annotations. For example, the 6 bp long *nad4L*-exon2 and the 11 bp long *cox1*-exon12 were not predicted by MFannot but they were resolved by Sanger sequencing of the cDNA clones. Such examples highlight the importance of verifying the structure of mt genes with cDNA sequences.

Manual curation of *nad4L* showed that its coding sequence overlaps by one base pair with the coding sequence of *nad5*, which is present just immediately downstream of *nad4L*. This is not unique, as the overlap of these two genes is found in other Leotiomycetes as well, including in *S. borealis*, *S. auriculariicola*, and *Antarctomyces pellizariae*. However, by using primers next to the start and stop codons of *nad4L* and *nad5*, we established that these two genes are expressed in *E. necator* from a single bicistronic transcript. Similarly, *atp6* and *nad3* are also side-by-side and are co-expressed in a bicistronic-like manner. To the best of our knowledge, co-transcription of the genes *nad4L*/*nad5* and *atp6*/*nad3* is rather exceptional among fungi^27^. Moreover, while in most fungal mt genomes, including those of several phylogenetically distant fungal species such as of members of the Sordariomycetes^11,13,28^, Leotiomycetes^16,21^, and Dothideomycetes^12,29,30^, the gene pairs *nad4L*/*nad5* and *nad2*/*nad3* are usually located close and next to each other, in *E. necator nad3* is paired with *atp6* instead of *nad2*. This is most likely the result of a gene rearrangement after divergence of the Erysiphales and a potential marker for powdery mildew pathogens. Furthermore, the widespread pairing among fungal mt genomes of *nad4L* and *nad3* to physically close genes raises the possibility that these genes require bicistronic-like behavior. One possible explanation for co-transcription of mt genes is that short mRNAs could be unstable or unable to interact effectively with the ribosomal unit^31,32^. For example, the coding sequences of *nad4L* and *nad3* in *E. necator* are relatively short, consisting of only 273 bp and 417 bp, respectively. However, this hypothesis does not account for *atp8*, which was not co-expressed with neighboring genes and has a coding sequence of only 147 bp. Nevertheless, future studies can shed light into the potential benefits and widespread behavior of bicistronic genes in mt genomes.

Sequencing of cDNA also revealed the presence of an *atp9* gene in the mt genome of *E. necator*. However, although transcribed, this gene is likely no longer functional because the encoded protein has several amino acids missing near the N-terminus, due to an in-frame stop codon present in its coding sequence. However, an *atp9* allele whose product can be translated into a full-length atp9 protein with an mt-targeting signal peptide at its N-terminus was identified in the nuclear genome of *E. necator.* This gene could be compensating for the inactive mt *atp9* allele, through allotopic expression in the nucleus and subsequent relocation of the produced protein into mitochondria. Indeed, allotopic expression of mtDNA-encoded genes that have migrated to the nucleus has been demonstrated in yeast (e.g., *atp8, bl4,* and *Var1p*)^33–35^ and human cell lines (e.g., *atp6* an*d atp8*)^36,37^. A study has also demonstrated the successful allotopic expression of the *Podospora anserina atp9* gene in the nucleus of *Saccharomyces cerevisiae*^38^, indicating that, as with other genes encoding ATP synthase subunits, *atp9* can at least in principle also be functionally expressed from nuclear DNA and its product is translocated in mitochondria. However, the same study also showed that an engineered nuclear version of the yeast *atp9* gene that contained an mt-targeting sequence was unable to compensate the function of the yeast mt *atp9* gene, indicating that there are barriers to the mt import of allotopically expressed proteins. Nonetheless, the presence of nuclear copies of *atp9*, which may or may not be accompanied by a parallel loss of the mt allele, have been reported in a number of fungal species^13,29,39^*,* including in the Leotiomycetes *Rhynchosporium* spp.^22^.

The 104 kb and 139 kb mt genomes of the barley powdery mildew *Blumeria graminis* f. sp. *hordei* isolates DH14 and RACE1, respectively, have been previously reported^40^. Compared to *E. necator*, the mt genome of *B. graminis* f. sp. *hordei* is considerably smaller but harbors all core mt genes in the same order and orientation as in *E. necator*. This indicates that their difference in mt genome size is likely due to presence/absence of nonfunctional regions, whereas no major mt gene rearrangement is present between them. Similar to *E. necator*, *B. graminis* f. sp. *hordei* also contains a nuclear-encoded *atp9* homolog that likely compensates for the absence of a functional mt-encoded *atp9*^40^. Notably, as in *E. necator*, the mt genome of *B. graminis* f. sp. *hordei* also contains the gene pair *atp6*/*nad3* next and physically close to each other, which indicates that this atypical gene pairing is common among powdery mildews.

Similar to previous reports of mt genomes of other members of the Leotiomycetes, such as *S. borealis*^21^ and *Monilinia laxa*^41^, the mt genome of *E. necator* is also enriched with ORFs encoding HEs and RTs. HE genes are selfish genetic elements that spread at a super-mendelian rate within a population. They are believed to have no effect on the fitness of the host organism, and therefore are not subject to natural selection. Once fixed in a population, these elements accumulate mutations that eventually disrupt their ability to spread^42,43^. However, comparative analysis among five isolates revealed that only six out of the 64 ORFs encoding HEs or RTs contained polymorphic sites, indicating that these ORFs have little genetic variability in the mt genome of *E. necator*. One possible explanation is that these enzymes could function as maturases required for proper intron splicing^44,45^. Mutations could disrupt their function, causing retention of introns in mature transcripts and interfering with the function of core mt genes. Future studies can reveal how active these enzymes are, their importance for the proper function of the mt genome of *E. necator*, as well as their distribution among different populations of powdery mildew pathogens.

## Methods

### Mt genome assembly

A scaffold (JNVN01000008.1) containing the mt genome of *E. necator* was initially identified by querying with BLASTn (e-value < 1e − 5) the mt genome of the phylogenetically close-related species *S. borealis* (NC_025200.1)^21^ against the nuclear genome assembly of *E. necator* C-strain (ASM79871v1)^2^. To produce the final mt genome of *E. necator*, a new assembly was generated in order to patch a 153 bp gap that was present within scaffold JNVN01000008.1. For this purpose, whole-genome sequencing (WGS) reads from *E. necator* isolate C-strain (SRR1448449) were obtained from NCBI. Reads were then trimmed with fastp v0.20^46^, and those with a *k*-mer matching to scaffold JNVN01000008.1 were extracted with the *bbduk.sh* script of the BBMap v38-72 software package^47^, using the parameters *k* = *31* and *hdist* = *1*. Extracted reads were then processed with the *bbnorm.sh* script of BBMap to normalize the depth of coverage to 100x, and the normalized reads were assembled into contigs with SPAdes v3.14^48^ utilizing *k*-mer values of 21, 33, 55, 77, 99, and 127. The assembled contigs were finally mapped to scaffold JNVN01000008.1 with the Burrows-Wheeler Aligner-Maximal Exact Matches (BWA-MEM; v0.7.17) algorithm^49^ and the gap was manually patched. Finally, the same reads utilized in the assembly step were mapped to the gap-filled contig with BWA-MEM v0.7.17 followed by two rounds of polishing with Pilon v1.23^50^.

### Annotation of mt genes

The assembled mt genome was initially annotated with MFannot, using the genetic code 4 (Mold, Protozoan and Coelenterate Mt Code)^51^. Genes encoding mt-tRNAs and their secondary structures were obtained with MITOS2^52^. Introns were classified into group I or group II with RNAweasel^15^. Intronic ORFs were identified with ORFfinder v0.4.3^53^, using as a minimum ORF length 200 bp and genetic code 4. ORFs encoding homing endonucleases (HEs) or reverse transcriptases (RTs) were identified and classified based on their conserved domains identified by querying (e-value < 1e − 3) the encoded peptide sequences against the NCBI conserved domain database (CDD)^54^. Conserved domains within introns were identified by translating the entire intronic sequences in six frames with the *transeq* script of the EMBOSS software package v6.6.0^55^, utilizing the genetic code 4 and querying (e-value < 1e − 3) the peptide sequences against the NCBI CDD. Codon usage was determined with the *cusp* script of the EMBOSS software package v6.6.0^55^. Short exact repeats were identified with REPuter^56^ using minimum repeat length of 8 bp and e-value < 1e − 5. Tandem repeats were identified with Tandem Repeat Finder v4.09^57^ and the overall percentage of repeats in the mt genome was calculated based on self BLASTn searches (e-value < 1e − 10), utilizing the parameter*-task blastn*. Circular representations of the mt genome was created with Circos v0.69-8^58^.

### Confirmation of mt genes by full length cDNA clones and Sanger sequencing

To validate the in silico annotations of protein-coding mt genes, RNA-seq reads of *E. necator* isolate C-strain were obtained from NCBI (accessions SRR1502871 to SRR1502882) and mapped to the mt genome with HISAT2 v2.2.1^59^. However, the overall low number of RNA-seq reads mapped prohibited curation of most of the *E. necator* genes. Therefore, a different approach was followed to adjust the annotations of the mt genes based on cDNA sequencing. To do so, *E. necator* C-strain was maintained on detached leaves of *Vitis vinifera* cv. Carignan in the laboratory as described before^2^. RNA was extracted from spores collected from colonized leaves by using the TRIzol reagent (Invitrogen, Carlsbad, CA). Complementary DNA was synthesized with the SuperScript First-Strand Synthesis Kit, (Invitrogen, Cat. no. 12371-019) according to the manufacturer’s protocol. Primers were designed to capture the entire ORF of genes (Supplementary Fig. S3, Supplementary Table S5) and utilized to PCR-amplify them from the cDNA template. For cloning, PCR products were separated on 2% agarose gel, bands were excised and subjected to column purification using the Zymoclean Gel DNA Recovery Kit (Zymo Research, Cat. no. D4001). Purified cDNA fragments were ligated into pGEM-T Easy vector (Promega, Madison, WI, United States) according to the manufacturer’s instructions and transformed into *Escherichia coli* strain DH5α, using the heat-shock method. Either PCR products or fragments cloned in the pGEM-T Easy vector were Sanger-sequenced. Generated ABI sequence files were mapped to the in silico predicted mt genes of *E. necator* with SnapGene v5.0.7 (GSL Biotech; available at snapgene.com). Alignments were visualized with SnapGene and exon–intron boundaries were manually adjusted. Start and stop codons were adjusted based on homology with reviewed fungal mt proteins from UniProt/Swiss-Prot database^60^.

### Phylogenetic analysis

To construct a phylogenetic tree of mt genomes, protein sequences were obtained from NCBI using the *efetch* module of the Entrez Direct package v13.9^61^. To construct a phylogenetic tree of nuclear genomes, universal single-copy genes were identified with BUSCO v4.0.6^62^ using the Eukaryote data set v10. Protein sequences were aligned with MAFFT v7.475^63^ and positions containing gaps in the alignments were removed with trimAl v1.4.1^64^. The resulting alignments were concatenated, thus producing alignments of 2651 and 51,776 amino acids for the mt and nuclear genomes, respectively that were subsequently used to construct trees with the Bayesian Inference method implemented in MrBayes v3.2.6^65^. Four chains were run with one cold and three hot for 500,000 generations, and sampling every 200 generations. The first 25% of samples were discarded as burn-in. The amino acid substitution model was set to *mixed*, which leveraged over 10 different models implemented in MrBayes, and subsequently selected *Cprev* and *Wag* as the best-fitted models for the mt and nuclear genomes, respectively. Stationarity was observed based on the average standard deviation of split frequencies, which was less than 0.005 at the end of the run. The trees were visualized and edited with FigTree v1.4.2^66^. Accession numbers of the proteins utilized to construct the phylogenetic trees are shown in Supplementary Tables S12 and S13.

### Comparison of mt genomes from different isolates of *E. necator*

Whole-genome sequencing reads of *E. necator* were downloaded from NCBI database for isolates e1 − 101 (SRR1448468), Branching (SRR1448453), Ranch9 (SRR1448454), Lodi (SRR1448470), and C-strain (SRR1448450). Reads were trimmed with fastp v0.20^46^ with default settings, except of the required read length that was set to 40 bp. Reads were then mapped simultaneously to the nuclear (GCA_000798715.1) and mt genomes of *E. necator* C-strain, using the BWA-MEM v0.7.17 software package^49^. Mapped reads considered as PCR duplicates were marked with samblaster 0.1.26^67^. The overall alignment rate and the number of reads that mapped to the mt and nuclear genomes were determined with SAMTools v1.9^68^. Coverage of the nuclear and mt genomes was determined with mosdepth v0.3.1^69^ with parameters adjusted to calculate the median coverage (option *-use-median*) and to ignore unmapped reads, secondary alignments and PCR duplicates (option *-flag 1796*). To avoid repetitive regions in the nuclear genome that can skew coverage values, mosdepth calculated the median coverage of all predicted exons in the nuclear genome. Subsequently, the nuclear genome coverage was estimated as the median coverage of all exons. The nuclear genome size of *E. necator* was estimated as the total number of sequenced bases from reads not marked as PCR duplicates that did not map to the mt genome divided by the nuclear genome coverage.

To identify polymorphic sites in the mt genome, reads had their coverage normalized to 100× with the *bbnorm.sh* script of the BBMap v38.18 software package^47^, and they were then mapped to the reference *E. necator* mt genome using the BWA-MEM v0.7.17 software package^49^. Short INDELs and SNPs were identified with FreeBayes v1.3.5 with ploidy set to 1^70^, and subsequently filtered with VCFtools v0.1.16 with minimum quality of 30^71^. Filtered INDELs and SNPs were annotated with SnpEff v5.0^72^ based on a database constructed from the GenBank file of the mt genome (MT880588). Polymorphic sites that overlapped with exons, introns or intergenic regions were identified with the subcommand *intersect* from BEDTools v2.29.0^73^.

## Supplementary Information

Supplementary Information.

Supplementary Tables.

## Data Availability

The annotated mt genome of *E. necator* has been submitted to GenBank under the accession number MT880588. Scripts used in the analysis were designed with the Snakemake workflow manager^74^, and are available at https://github.com/alexzaccaron/2021_enec_mt.
