# Supplementary material for: The mitochondrial genome of the grape powdery mildew pathogen Erysiphe necator is intron rich and exhibits a distinct gene organization
Source: Sci Rep. 2021 Jul 6;11:13924. doi: 10.1038/s41598-021-93481-5 (PMC8260586; doi:10.1038/s41598-021-93481-5)
Supplement: Supplementary file 1 — Supplementary Information. [file 41598_2021_93481_MOESM1_ESM.docx]

**Supplementary Materials**

Title: **The mitochondrial genome of the grape powdery mildew pathogen *Erysiphe necator* is intron rich and exhibits a distinct gene organization**

Authors/Affiliations: Alex Z. Zaccaron^a^, Jorge T. De Souza^a,1^, and Ioannis Stergiopoulos^a*^

^a^ Department of Plant Pathology, University of California Davis, Davis, CA, USA.

*Correspondence: Ioannis Stergiopoulos: University of California Davis, Department of Plant Pathology, One Shield Avenue, Davis, CA 95616-8751, USA, Tel: +1-530-400-9802, email: istergiopoulos@ucdavis.edu.

^1^ Permanent Address: Federal University of Lavras (UFLA), Department of Plant Pathology, 37200-000 Lavras, MG, Brazil

**Supplementary Tables**

(Supplementary Tables are provided in a separate excel file)

**Table S1.** Statistics of fungal mitochondrial genomes publicly available. Genomes were sorted by length. *Erysiphe necator* is highlighted. These mitochondrial genomes were found in the NCBI Organelle database

(https://www.ncbi.nlm.nih.gov/genome/organelle/) as of January 2020.

**Table S2.** Codon usage of the core mitochondrial genes from *Erysiphe necator*. The table shows the total number of codons for each gene. The last two columns show the number of GC bases for each codon and usage fraction among synonymous codons.

**Table S3.** Repeats identified with REPuter in the mitochondrial genome of *Erysiphe necator*. Repeats were identified with REPuter (https://bibiserv.cebitec.uni-bielefeld.de/reputer) at E-value < 1e-5 and minimum repeat length of 8 bp.

**Table S4.** Tandem repeats identified in the *Erysiphe necator* mitochondrial genome. Start and end coordinates in the genome are shown as well as the predicted copy number. The repeats were identified with the Tandem Repeat Finder program (<https://tandem.bu.edu/trf/trf.html>).

**Table S5.** Primers utilized to amplify and sequence the core mitochondrial genes of *Erysiphe necator*. The location of the primers in the mature gene transcripts are shown in Supplementary Fig. S3.

**Table S6.** Mitochondrial atp9 genes in members of Leotiomycetes and other fungal species. In *Erysiphe necator* as well in *Scytalidium auriculariicola* and *Botrytis cinerea* (highlighted in green), atp9 is present but encodes a truncated protein.

**Table S7.** Classification of *Erysiphe necator* mitochondrial introns and of the conserved domains encoded by their sequences. Introns start and end coordinates in the mt genome of *E. necator* are shown. Conserved domains found within the introns are shown. Conserved domains with more than one copy are indicated in the last column.

**Table S8.** Intronic open reading frames (ORFs) encoding homing endonucleases (HEs) and reverse transcriptases (RTs) in the mitochondrial genome of *Erysiphe necator*. ORF IDs are composed of the gene name and the intron number (5’ to 3’ direction) where the ORFs are inserted. ORFs encoding proteins containing conserved domains related to RT (pfam00078) and HEs of the families LAGLIDADG_1 (pfam00961), LAGLIDADG_2 (pfam03161) and GIY-YIG (cd10445) are indicated. Combination of more than one domain is indicted with a plus sign. The length of the domains (in amino acids) is also indicated, when present. ORFs were considered degenerated if the length of the conserved domains is shorter than the expected. For each intronic ORF, the phase of the preceding, i.e., upstream exon is given in relation to the start codon (phase = 0) of the respective gene. The phase of the ORF is in relation to the previous exon, i.e., ORFs in-frame with the previous exon have phase = 0. Finally, the distance in base pairs between the start of the ORF and the end of the previous exon is shown as well as the number of in-frame stop codons (TAA and TAG) in these regions.

**Table S9.** Polymorphic sites identified in the mitochondrial genes of *Erysiphe necator*. For each polymorphic site, its position in the genome is shown (C-strain as reference; GenBank MT880588.1) as well as the alleles of the reference genome (referred to as allele 0) and alternative alleles (referred to as alleles 1, 2 or 3, separated by commas). Alleles of four other *E. necator* isolates (Lodi, E1-101, Branching, and Ranch9) are indicated. Polymorphic sites located within functional regions of the genome are highlighted. Further information of these sites are given in the form of the gene affected, which can be an intron encoded ORF (LD: LAGLIDADG, RT: reverse transcriptase) or a conserved mitochondrial gene; variants at the DNA and protein levels; and a description of the mutation.

**Table S10.** Overrepresentation of the mitochondrial (mt) genome of different *Erysiphe necator* isolates in whole-genome sequencing (WGS) reads. Reads were mapped to the mt and nuclear genomes simultaneously.

**Table S11.** Estimation of mtDNA copy number and size of the nuclear genome of five isolates of *Erysiphe necator*. Only reads that passed quality control (QC) and were not flagged as PCR duplicates (SAM Flag 1024) were considered for the calculations. The coverage of the nuclear genome was determined as the median coverage of exons from nuclear genes (GCA_000798715.1), whereas coverage of the mt genome was calculated as the median coverage of all bases in the mt genome. The estimated mtDNA copy number was calculated as the ratio between the mt genome coverage and the nuclear genome coverage. The estimated nuclear genome size was calculated as the total base pairs from reads after QC, not flagged as PCR duplicates, that did not map to the mt genome, divided by the nuclear genome coverage.

**Table S12.** GenBank accession numbers of genes utilized to construct a phylogenetic tree of mitochondrial genomes.

**Table S13.** GenBank accession numbers and sequences of genes utilized to construct a phylogenetic tree of nuclear genomes. Protein sequences from a total of 113 genes universally conserved in Eukaryotes were used to infer a tree. Genes were identified with BUSCO v4.0.6 using eukaryota_odb10. BUSCO was executed in genome mode for genomes without gene annotation. Protein accession number or sequence is shown for each BUSCO gene. BUSCO gene IDs are shown in the headers of the last 113 columns. BUSCO genes for *Scytalidium auriculariicola* and *Rhynchosporium orthosporum* were not predicted because they have no reference nuclear genome assemblies available at NCBI.

**Supplementary Figures**


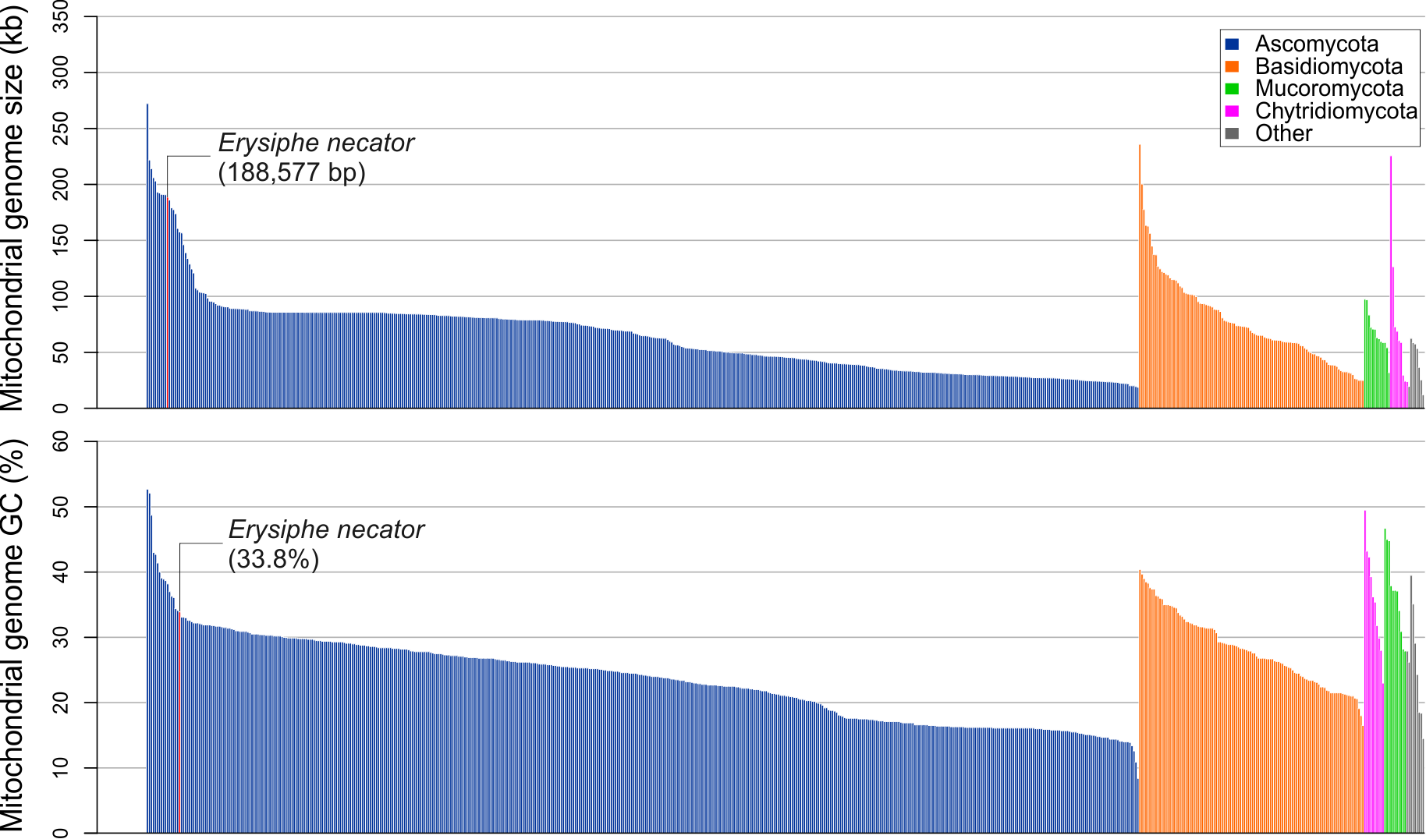


**Fig. S1.** Comparison of size and GC content of 636 fungal mitochondrial (mt) genomes. Each mt genome is represented by a single bar in the graph and is color-coded based on the phylum that the respective species belongs; blue: Ascomycota, orange: Basidiomycota; green: Mucoromycota, purple: Chytridiomycota, grey: Zoopagomycota, Blastocladiomycota and Cryptomycota. The bar representing the mt genome of the powdery mildew pathogen *Erysiphe necator* is highlighted in red. Mt genomes were organized by phylum and then by size (top bar chart) and GC content (bottom bar chart). The complete list of the fungal mt genomes included in these bar graphs can be found in Supplementary Table S1.


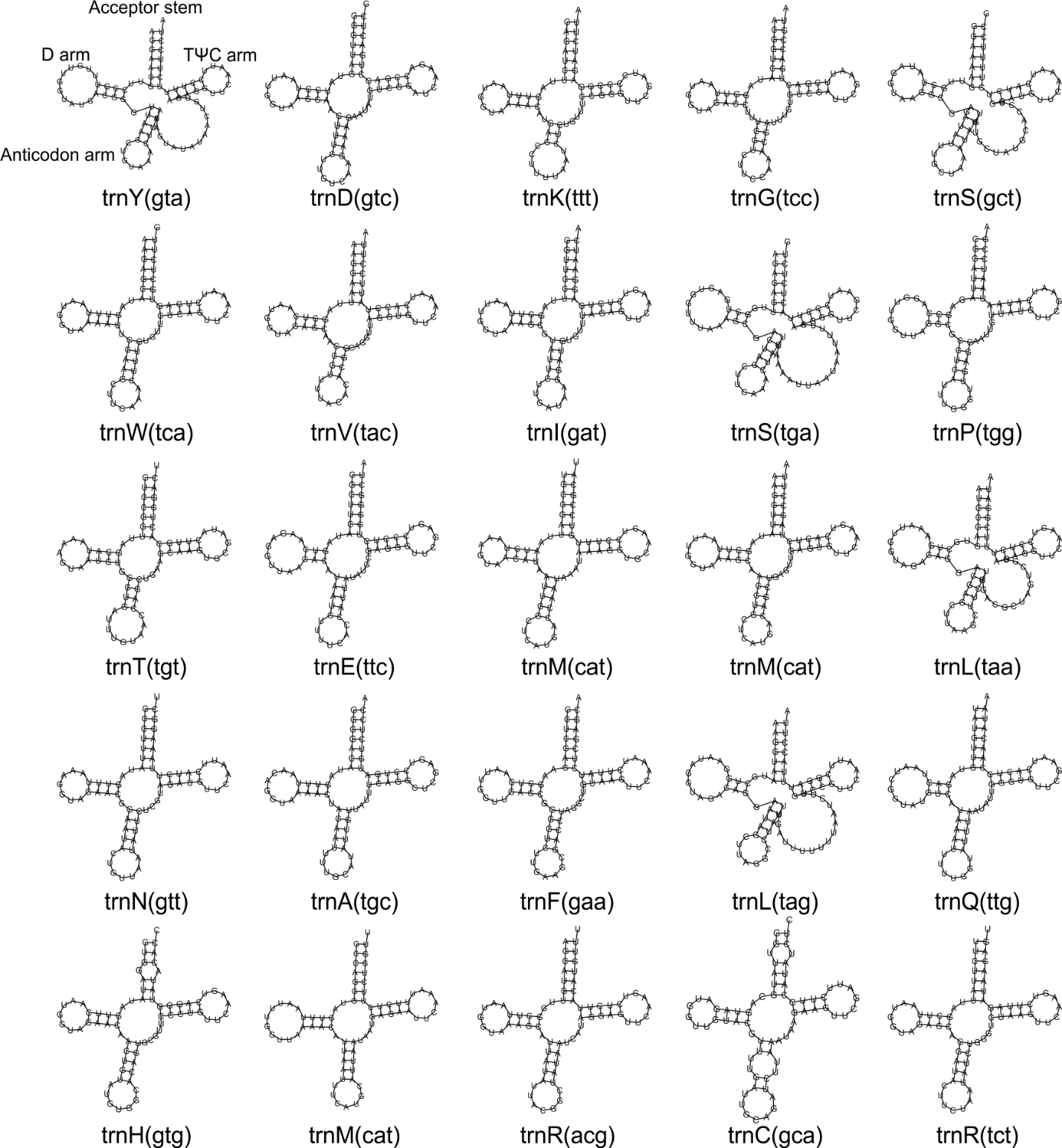


**Fig. S2.** Predicted secondary structures of mitochondrial tRNAs of *Erysiphe necator*. The 25 tRNAs are arranged in the figure top to bottom, left to right, according to their order of occurrence in the mitochondrial genome. The tRNA arms are illustrated for trnY(gta). Respective tRNA-anticodons are shown between parentheses. Structures of tRNAs were predicted with MITOS2 web server (<http://mitos2.bioinf.uni-leipzig.de/index.py>).

**
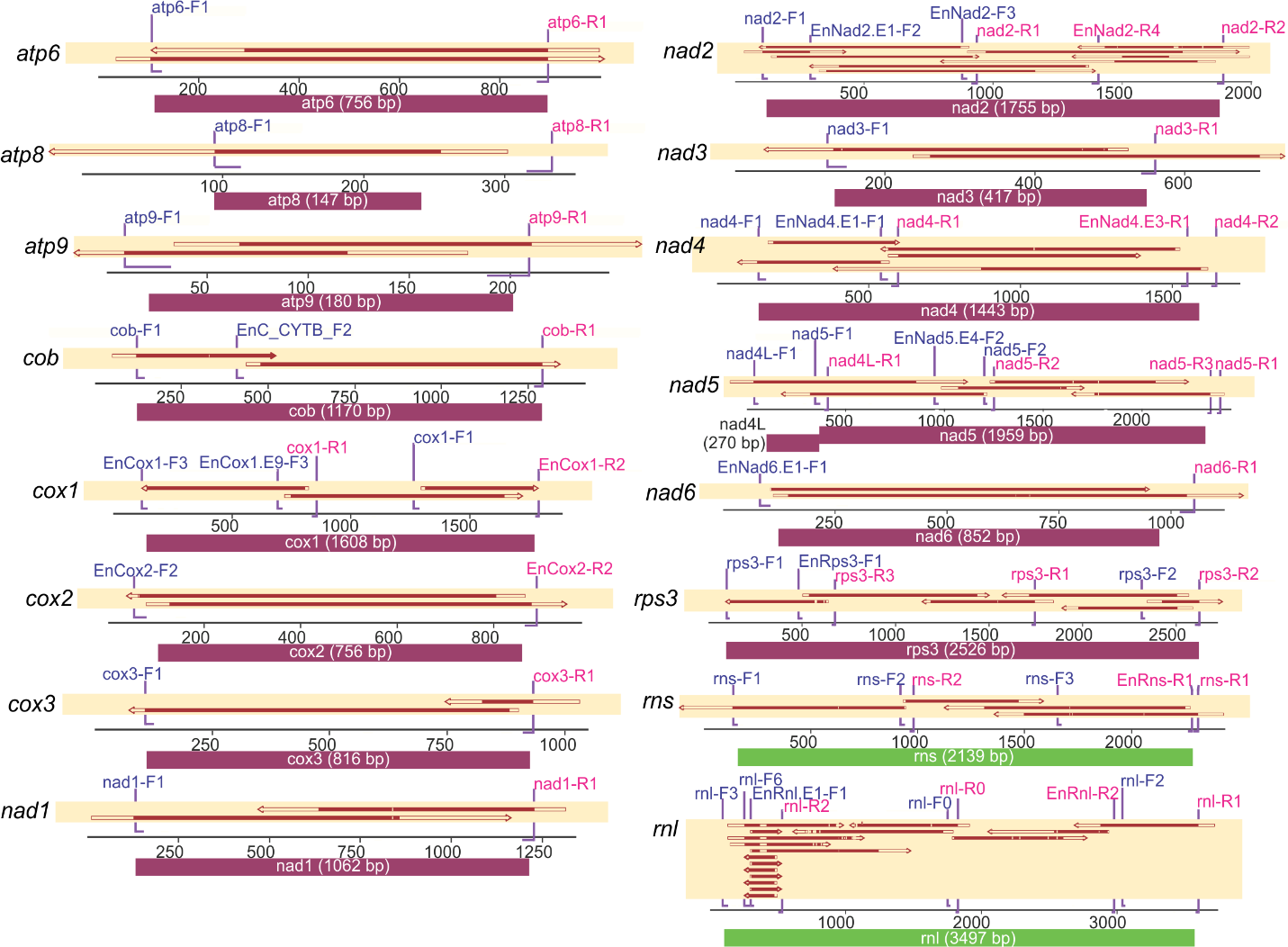
**

**Fig. S3.** Manual verification and confirmation of *Erysiphe necator* mitochondrial genes. Thick dark red and green bars represent coding sequences (exons for rRNAs). Thinner arrows indicate coverage of sequenced PCR products. Within these thin arrows, filled regions represent matches to the coding (or exonic) sequence, while transparent regions indicate mismatches or gaps in the sequenced PCR product. Deletions in the reference sequence are represented by red triangles (no occurrences). Location of primers are indicated with thin horizontal purple lines above (forward primers, in blue) or below (reverse primers, in pink) the scale bar. Primer sequences are shown in Supplementary Table S5. Gene names are indicated. Figures were generated with SnapGene v5.0.7 (https://www.snapgene.com).


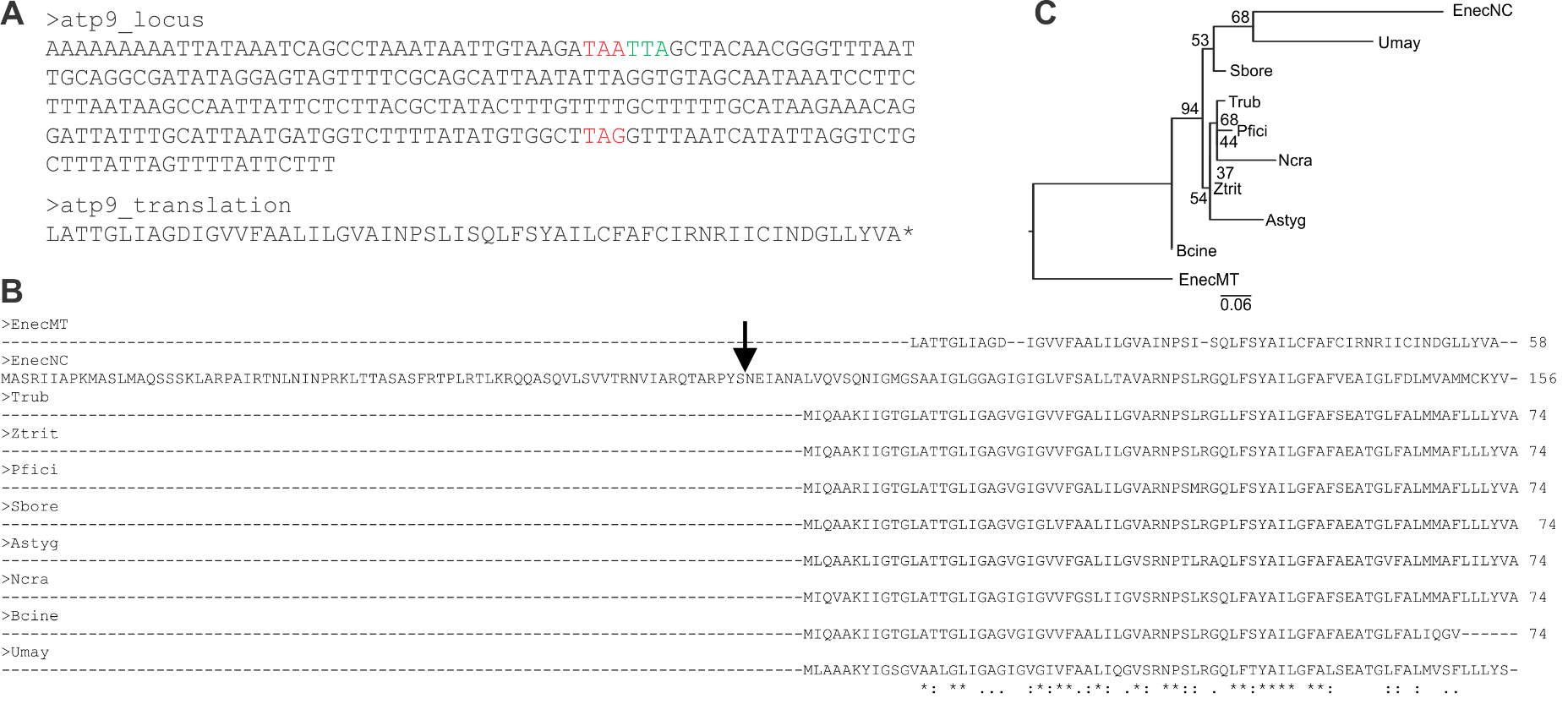


**Fig. S4.** The mitochondrial ATP synthase subunit 9 (*atp9*) gene is likely a pseudogene in *Erysiphe necato*r. (A) Nucleotide sequence of the truncated mitochondrial (mt) *atp9* gene of *E. necator* and its translated 59 amino acid product based on the Mold, Protozoan, and Coelenterate mt genetic code (genetic code = 4). Predicted start and stop codons are highlighted in green and red, respectively. The three nucleotides immediately before the predicted start codon are also highlighted and encode a different stop codon. (B) Amino acid alignment of the mt ATP synthase subunit 9 encoded by the mt (EnecMT) and nuclear (EnecNC) *atp9* genes of *E. necator*. Included in the alignment are also homologous atp9 proteins from *Botrytis cinerea* (Bcine; AGN49018), *Zymoseptoria tritici* (Ztrit; YP_001648752), *Trichophyton rubrum* (Trub; Q01554), *Pestalotiopsis fici* (Pfici; AOW71154), *Neurospora crassa* (Ncra; Q12635), *Annulohypoxylon stygium* (Astyg; AYE67549), *Sclerotinia borealis* (Sbore; YP_009072368), and *Ustilago maydis* (Umay; Q0H8W9). The predicted cleavage site of the mt signal present in the ATP synthase subunit 9 encoded by the nuclear *atp9* gene of *E. necator* (KHJ33827) is indicated by an arrow. The alignment shows that the EnecMT atp9 misses a few amino acids in its N-terminus and a few others that are fairly conserved among fungal atp9 proteins. (C) Maximum likelihood phylogenetic tree constructed based on the amino acid alignment of selected atp9 proteins presented in Panel B. The tree was inferred with IQ-TREE v1.6.11 utilizing the cpREV amino acid substitution model, selected automatically by IQ-TREE as the best model, and 1,000 ultra-fast bootstrap replicates. The tree shows that the atp9 protein encoded by the mt *atp9* gene of *E. necator* strains apart from other fungal atp9 proteins.

**
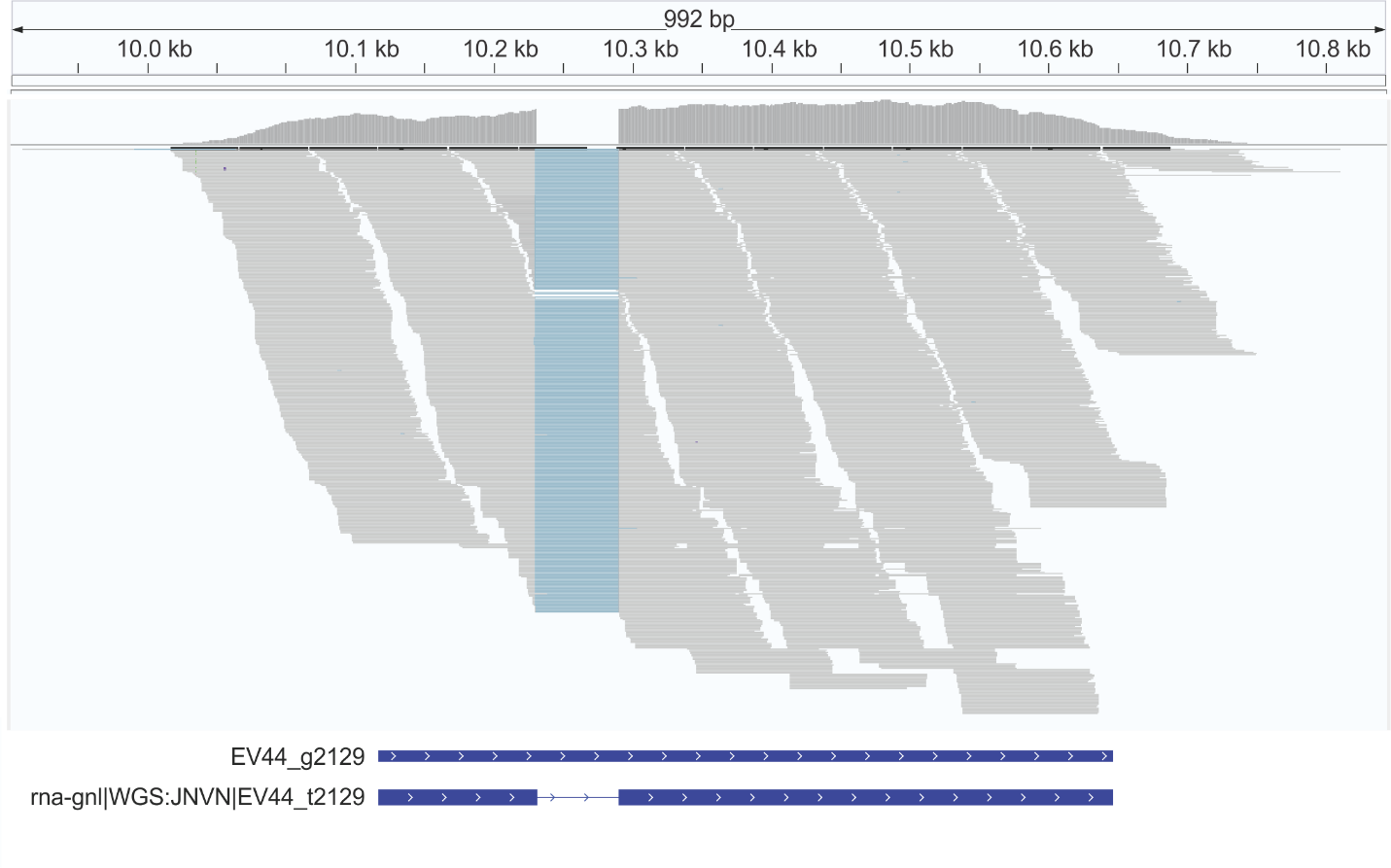
**

**Fig. S5.** The ATP synthase subunit 9 (*atp9*) gene encoded in the nuclear genome of *Erysiphe necator* is expressed. RNA-seq coverage at the region of the *E. necator* C-strain nuclear genome encoding a putative ATP synthase subunit 9 (GenBank accession KHJ33827). Gene structure is shown in blue at the bottom. A total of 3,317 RNA-seq reads mapped to the region and are shown in grey (GenBank SRA accession SRR1502880). Region was visualized with IGV v2.6.1 (https://igv.org).


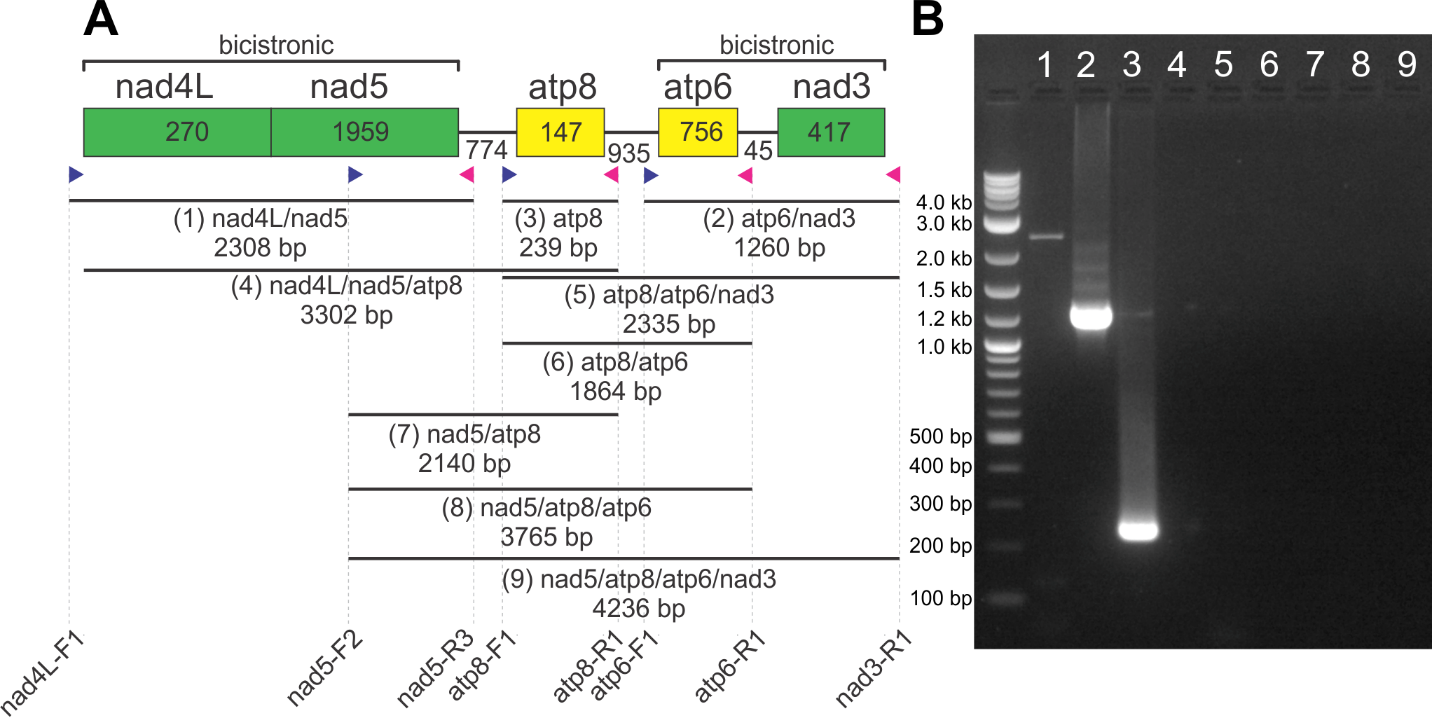


**Fig. S6.** Co-transcription of the mitochondrial gene pairs *nad4L*/*nad5* and *atp6*/*nad3* in *Erysiphe necator*. (A) Schematic representation of the organization of the *nad4L*, *nad5*, *atp8*, *apt6*, and *nad3* genes in the mitochondrial (mt) genome of *E. necator*. Length (bp) of coding sequences (i.e., with introns spliced out) are shown inside boxes that represent each gene. Lengths in base pairs of intergenic regions are shown in between boxes. Location of primers in the coding sequences are indicated with triangles. Horizontal lines indicate distances (bp) between primers. Introns were not considered when calculating these distances. Primer sequences are shown in Table S5. (B) Agarose gel electrophoresis of PCR products depicting co-transcription of the mitochondrial gene-pairs *nad4L*/*nad5* and *atp6*/*nad3* in *E. necator*. cDNA of isolate C-strain was used as template in PCRs with primers aimed to detect bicistronic or polycistronic expression among the *nad4L*, *nad5*, *atp8*, *apt6*, and *nad3* genes. The nine primer combinations used (1-to-9) and the expected size of the products are shown in panel A. The exact location of the primers is shown in Fig. S3. Amplification of *atp8* was used as a positive control for the PCRs.


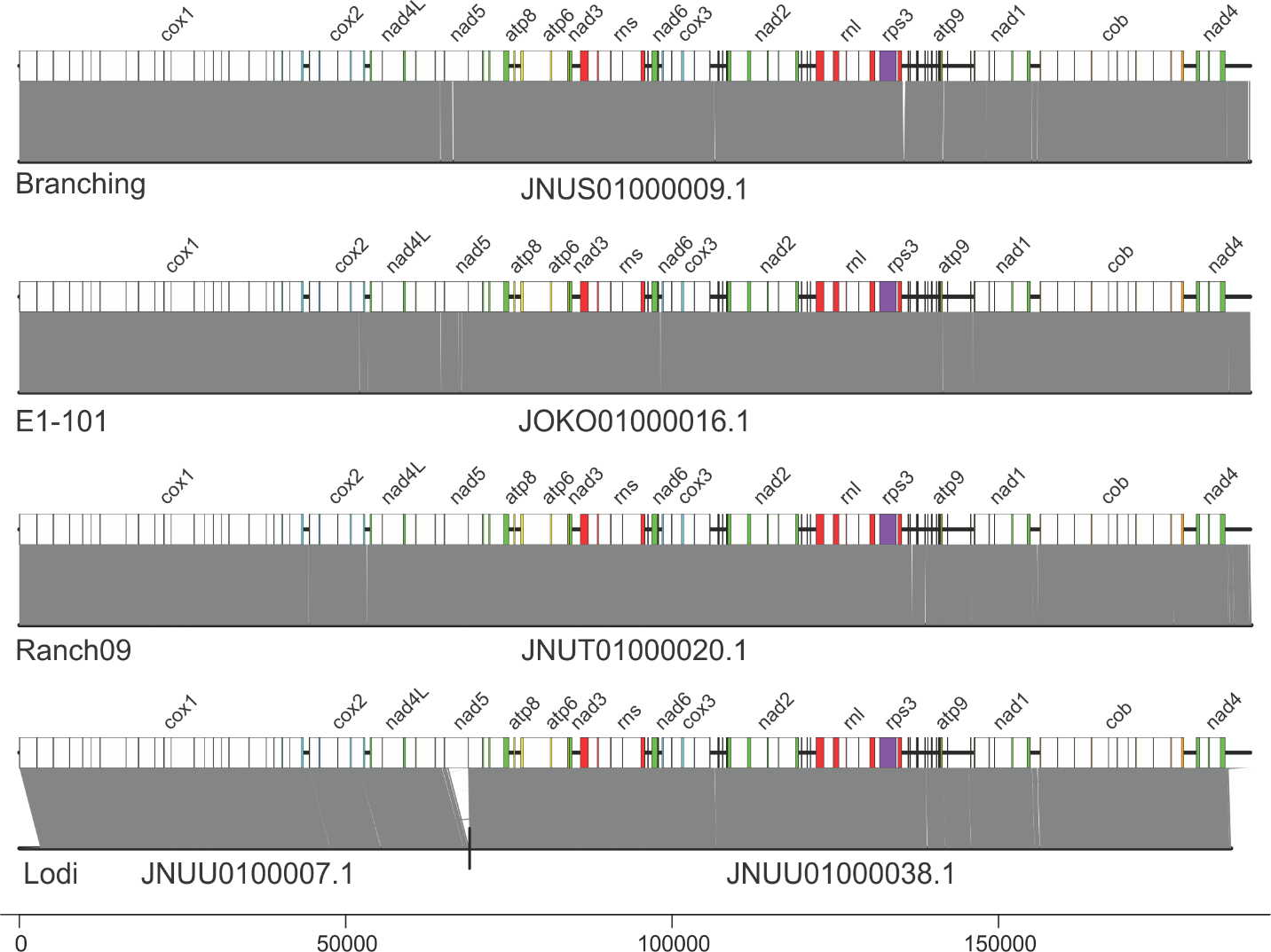


**Fig. S7. The mitochondrial genomes of different isolates of *Erysiphe necator* are highly conserved.** (A) Homologous regions between the mitochondrial (mt) genome of the *E. necator* C-strain (top strand) and the assembled scaffolds (bottom strand) containing the mt genomes of isolates Branching, Ranch9, e1-101, and Lodi. GenBank accession numbers of the contigs are show at the bottom. Mt genes of *E. necator* C-strain are represented as rectangles with intronic regions shown in white. Gray ribbons, seen as grey blocks in the figure due to their density, represent homologous regions identified with BLASTn searches with e-value < 1e-20. Scaffolds were rotated in order to match the start position of the mt genome of C-strain. The mt genome of strain Lodi is fragmented into two scaffolds, indicated by a vertical line at position 68,980. Intron 4 of gene *nad5* in isolate Lodi is missing in the assembled scaffolds. However, the region of this intron had normal coverage when whole-genome sequencing reads from isolate Lodi were mapped to the reference mt genome (Fig. 3), indicating that this intron is conserved in isolate Lodi.

**Fig. S8: Comparison of the mitochondrial (mt) and nuclear genome phylogeny of different fungi.** Bayesian phylogenetic trees were constructed based on the mt and nuclear genomes, and are shown on the left-hand and right-hand side, respectively. The mt genome tree was constructed based on the concatenated alignment of the protein sequences of 12 core mt genes (*atp6*, *nad1*-*6*, *nad4L*, *cox1*-*3,* and *cob*). The nuclear genome tree was constructed based on the concatenated alignment of the protein sequences of 113 universally conserved genes in Eukaryotes. Supporting values of branches are indicated as Bayesian posterior probabilities. *Morchella importuna* (Pezizomycete) was used as outgroup in both trees. To construct the nuclear genome tree, universally conserved genes were identified with BUSCO v4.0.6 using the Eukaryote data set v10, which contains a total of 255 genes. For species with gene annotation available at NCBI, BUSCO was executed in proteome mode (--*mode proteins*). For species with no gene annotation available at NCBI, BUSCO was executed in genome mode (*--mode genome*). Species with no nuclear genome assembly available at NCBI were not included in the nuclear genome tree. All complete BUSCO genes (n=113), i.e., not duplicated or fragmented, in all analyzed species were utilized to infer the tree. Both trees were constructed with MrBayes v3.2.6 using the same parameters (see Methods in the main manuscript). Accession numbers of the proteins used to construct the trees are shown in Supplementary Tables S12 and S13 for the mt and nuclear genome trees, respectively.
